# Comparison of three sampling methods for small-bodied fish in lentic nearshore and open water habitats

**DOI:** 10.1007/s10661-021-09027-9

**Published:** 2021-04-09

**Authors:** Joseph E. Merz, Jesse T. Anderson, Jesse Wiesenfeld, Steven C. Zeug

**Affiliations:** 1 Cramer Fish Sciences, 3300 Industrial Blvd., Suite 100, West Sacramento, CA 95691 USA; 2 Department of Ecology and Evolutionary Biology, University of California Santa Cruz, 130 McAllister Way, Santa Cruz, CA 95060 USA

**Keywords:** Gear comparison, Sampling Platform, Push net, Fish sampling, Monitoring, Lentic fish community

## Abstract

We performed a preliminary evaluation of a mobile sampling platform with adjustable push net and live box (Platform) against two common methods for sampling small-bodied fish (i.e., 10–100 mm) in two distinct lentic habitats. Nearshore (NS) littoral habitat was sampled by Platform and beach seine, and open water (OW) pelagic habitat by Platform and Kodiak trawl. Our goal was to evaluate the Platform’s ability to describe fish assemblage structure across habitat types in contrast to common techniques restricted to single habitat types that are less comparable due to gear-specific bias. Platform sample speed had a significant positive effect on recapture efficiency of both nearly neutrally buoyant objects and marked fish. Marked fish recapture efficiencies were similar for Platform in NS and OW, indicating similar efficiency across habitat types. Platform capture efficiency was similar to beach seine and greater than Kodiak trawl. With similar sampling time, the Platform collected more individuals and taxa in NS relative to beach seine and in OW relative to Kodiak trawl. Greater taxa detection by the Platform suggests that it may be effective at detecting species that are numerically rare in specific habitats when compared to these methods. Fish CPUE was significantly greater NS regardless of technique. However, by using the Platform, there is greater confidence that this difference was reliable and not a gear selectivity artifact. Overall, this preliminary study demonstrates the Platform’s potential to collect standardized data across NS and OW habitats, track ontogenetic habitat shifts, and detect differences in small-bodied fish taxa richness, relative abundance, and density between NS and OW habitats. Continued experimentation beyond a single reservoir and fish size range is required before consensus can be established regarding the utility of this new push net design.

**Supplementary information:**

The online version contains supplementary material available at 10.1007/s10661-021-09027-9.

## Introduction

Research on fish-based indices of biological integrity (e.g., Karr, 1981) has greatly improved fish monitoring methods and techniques, broadening our understanding of factors structuring occurrence, abundance, and composition of inland fish assemblages (e.g., Angermeier & Smogor, 1995; Aparicio et al., 2011; Deegan et al., 1997; Lyons, 1992). However, the inherent variability of many aquatic environments, coupled with gear-specific limitations and habitat-specific sampling methodologies have made it difficult to standardize and compare monitoring and research across a range of freshwater and estuary habitats (Jurajda et al., 2009; Poff & Allan, 1995; Revenga et al., 2005).

Lentic systems are particularly difficult to accurately survey because they have distinct physicochemical zones, often requiring multiple sampling methods (Coates et al., 2007; Fischer & Quist, 2014a, 2014b; Idelberger & Greenwood, 2005). Use of disparate sampling techniques has led to research focused on describing fish assemblages within individual zones (e.g., limnetic: McQueen et al., 1986; Gido & Matthews, 2000; littoral: Weaver et al., 1993; Ruetz et al., 2007) rather than whole-waterbody assemblages based on representative sampling with a single gear type across ecotones. Therefore, specific sampling gears are often selected based on habitat characteristics, such as depth, sampling area, water temperature, methodology limitations, or target species characteristics (e.g., Pierce et al., 1990; Bonar et al., 2009; Baran et al., 2017). For instance, the beach seine is commonly used to collect fish from a diversity of standing and flowing waters and is widely used to support research and monitoring for small-bodied fishes (Bonar et al., 2009; Mandrak et al., 2006; Poos et al., 2007). However, seine efficacy has been reported as relatively higher in dense macrophyte cover, lower over boulders or snags than level areas, and lower for benthic than midwater fishes. Beach seining is also limited by depth and samples relatively small water volumes per haul (LaPointe et al., 2006; Lyons, 1986; Pierce et al., 1990). Alternatively, trawls can sample relatively large open water volumes over a short period and have been commonly used to collect small-bodied fishes in open lentic fresh and estuarine waters (Feyrer et al., 2015; Herzog et al., 2009; Reid & Dextrase, 2017; Vorwerk et al., 2008). However, trawls are comparatively inefficient, lack maneuverability, and can alter fish behavior, making them inappropriate for sampling shallow, complex lentic habitats (Engås et al., 1998; Kaartvedt et al., 2012). Furthermore, trawls have been associated with fish injury and their efficiency is often difficult to test (Davis, 2002). Thus, composition of captured fish assemblages depends on gear used and sampling site conditions, limiting our ability to standardize sampling and interpret results across habitat types (Eggleton et al., 2010; Fischer & Quist, 2014a, 2014b).

Lack of standardized lentic sampling methods has also diminished our ability to evaluate temporal variation in fish abundance and habitat use (Guy & Willis, 1991; Pope & Willis, 1996; but see Bonar et al., 2009). Lentic freshwater and estuary environments are commonly sampled with different gears at specific times of year due to temporal shifts in habitat use (e.g., summer offshore fish movement, spawning) and variable fish recruitment to different sampling gears. For instance, young cohorts of small-bodied species that hatch in spring may not be susceptible to standard methods until the following year, whereas age-0 large-bodied species hatched during spring may be collected during their first fall (Fischer & Quist, 2014a, 2014b). Furthermore, because sensitive early life stages of many fish species require off-channel or vegetated littoral habitats, these life stages are often under-sampled due to limitations of traditional monitoring techniques, restricting our ability to track how environmental conditions influence ontogenetic habitat shifts (Rozas & Minello, 1997; Sommer et al., 2011). Therefore, standardized sampling across habitats, especially open water (pelagic) and shallow, complex, nearshore (littoral) habitats, is particularly important for accurate biological assessment (Bonar et al., 2009; Fisher & Quist, 2014a).

Thus, the need for standardization of lentic habitat sampling requires development of maneuverable methods that minimize sample area disturbance, quickly sample relatively large water volumes, and feasibly sample in and across shallow, complex, and open water habitats, while adequately characterizing richness, relative abundance, and presence of taxa that are numerically rare (i.e., < 1% relative abundance) in the habitat being sampled. To meet these challenges, we developed an integrated and mobile concentrator net and live box prototype, the Single-Platform Aquatic Species and Habitat Sampling System (Platform; US Patent No. 9,776,692 and 10,259,541).

We conducted a preliminary study using the Platform to compare species richness, relative abundance, and size distribution of small-bodied fishes between lentic shallow nearshore (NS; Bowen et al., 2003) and deep, open water (OW; Seitz et al., 2006) habitats as well as capture efficiency of nearly neutrally buoyant objects and marked fish. Our goal was to compare Platform catch data to those collected by two gear types commonly employed to sample each habitat separately [beach seine (Hahn et al., 2007) in NS, and Kodiak trawl (Damon, 2016) in OW habitats] to assess the ability of a single technique (Platform) to provide comparable data across habitats. In this preliminary study to assess the practicality of continued development of this novel sampling method, we focused on small-bodied fish and juveniles of select larger fish (i.e., 10–100 mm) that may or may not transition between NS and OW lentic habitats against two common methods used in freshwater and estuarine environments. We hypothesized that the Platform can be used to quantitatively compare small-bodied fish assemblage structure across NS and OW lentic habitat types where two less comparable methods would usually be required.

## Methods and materials

### Study location

**Fig. 1:**
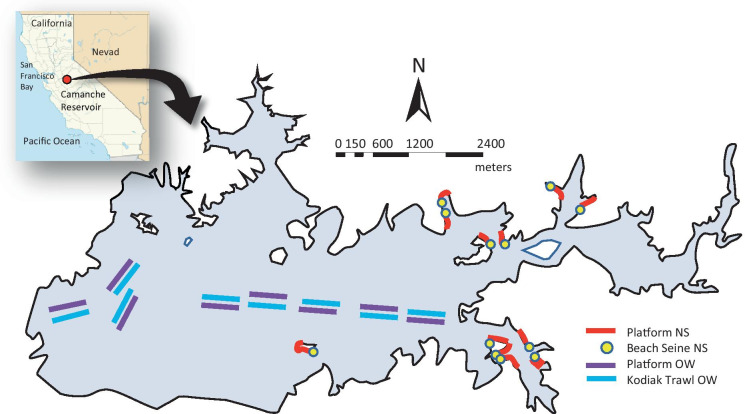
Sampling locations for beach seine and Platform at six shallow nearshore (NS) habitat sites (1A, 1B, 2A, 2B, 3A, and 3B), and Platform and Kodiak trawl at five open water (OW) sites (1–5) in Camanche Reservoir (far right) in relation to the entire reservoir (lower left) and California (upper left)

Sampling was conducted in eutrophic Camanche Reservoir (z_mean_ = 17 m; z_max_ = 31 m; v = 5.1 × 108 m^3^; 3100 ha) in the California western foothills of the Sierra Nevada Mountains (38° 13′ 11.26″ N, 120° 58′ 53.32″ W; Fig. 1). The reservoir is located on the Mokelumne River at the junction of Amador, Calaveras, and San Joaquin counties and is impounded by Camanche Dam (Beutel & Horne, 1999). Camanche Reservoir was selected because it has several ubiquitous fish taxa that transition between NS and OW and are found in multiple freshwater habitats (e.g., lakes, reservoirs, estuaries, and large rivers) throughout North America due to their transport and inoculation (e.g., clupeids, centrarchids, and cyprinids; Marchetti et al., 2001).

### Data collection

**Fig. 2:**
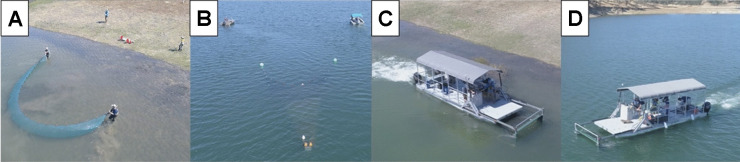
Images depicting **a** beach seine, **b** Kodiak trawl, and Platform in **c** nearshore (NS) and **d** open water (OW) habitats

Three sampling gear types were deployed on 23 May and 16, 26, 27 June 2016 and 5, 14 June 2017 (beach seine: NS; Kodiak trawl: OW; Platform: NS and OW). A 15.3 m by 1.2 m beach seine (3 mm mesh) was used to make hauls following the methods of Merz et al. (2016; Fig. 2a). For each haul, the beach seine was deployed parallel to shore on a perpendicular transect extending ~ 15.3 m from water’s edge or to a water depth of 1.2 m, whichever came first, and then retrieved to shore. Depth was recorded at three points along each transect: (1) maximum distance from shore, (2) half distance from shore, and (3) at shore. Water volume sampled was calculated by multiplying the number of linear meters traveled per haul by averaged seine beginning and end width and averaged depths recorded for each haul. The seine crew consisted of four people, including two guiding the seine bridles and two pulling the seine to shore with ropes. Each seine haul took approximately 23 min to sample, retrieve, and process fish.

The Kodiak trawl net varied from 51 mm stretch mesh at the net wings to 6 mm at the cod end and a fully expanded mouth opening of 1.8 m by 7.6 m (Fig. 2b). A 1.8 m bar was attached to the front of each wing with lead and float lines on the bottom and top of the net, respectively. The trawl typically sampled from the water surface to approximately the maximum net depth (~ 1.8 m). The Kodiak trawl incorporated a live box attached to the net cod end to reduce fish mortality. Actual trawl net fishing dimensions varied depending on boat speed and position and have been described in past reports (e.g., USFWS, 1995). Estimated Kodiak trawl net mouth size was 12.5 m^2^. The Kodiak trawl consisted of five people, including two on the trawl boat and three on the chase boat. Each 10-min trawl took approximately 35 min to set, sample, retrieve and process fish.

**Fig. 3:**
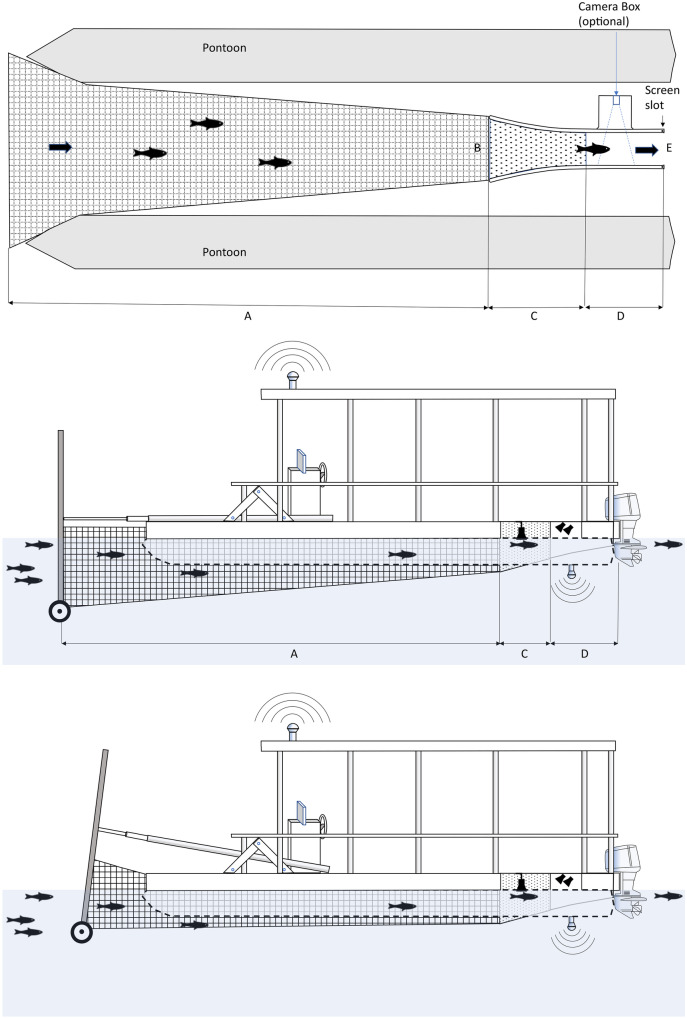
Diagram of the single-platform aquatic species and habitat sampling system (Platform). Top: vertically adjustable fyke net (surface to 3 m) fits between two pontoons (transparent in illustration to show live box) and terminates at live box near vessel aft where video images, captured fish, and water quality parameters are recorded. Wheel at bottom of rigid net frame allows rolling over benthos in shallow water. Bottom: top-down diagram of Platform (work deck removed) displaying the concentrator push net (**A** length = 5 m), and live box [**B** opening = 68.6 cm wide; **C** perforated aluminum (6 mm holes) section = 105.9 cm long; **D** solid plate section = 83.5 cm long, and **E** live box exit = 32.9 cm wide]. The screen is mounted into slot for active fish capture but can be removed for passive flow through in conjunction with optional camera box (not used in this study)

The Platform was designed to simultaneously collect fish and an array of coincident biotic and abiotic data in a variety of habitats (Figs. 2c, d and 3). It was a modified pontoon boat with a custom-built concentrator push net attached at the front of the boat that runs between two 7.3 m pontoons. A removable 1.9 m by 0.3 m live box was mounted underneath the boat’s deck where sampled water and associated fish can be accessed during operation (Fig. 3). The net was approximately 5.5 m long and was composed of two mesh size sections, 38 mm toward the mouth, and 6 mm toward the cod end. The rigid net mouth was 2.4 m wide by 1.2 m high and was hydraulically controlled from the helm to lower and raise the net mouth depending on desired sampling depth. The current prototype effectively samples from depths of approximately 0.4 to 3 m from the water surface. A rubber wheel on each bottom corner of the rigid frame allowed the frame to roll along the lakebed in shallow water. Shear pins at the base of the rigid frame attachment to the hydraulic arms prevented equipment damage if the frame struck solid structures (e.g., bedrock, logs, etc.).

The live box consisted of two sections. The first was constructed of perforated aluminum (6 mm holes on 4.76 mm staggered centers; 40% open area) and attached to the net. The second section was constructed of solid aluminum plate (8 gauge) and had a detachable back screen to allow fish capture or passive monitoring (e.g., flow through). The Platform was propelled by two independently controlled 50 hp outboard motors, providing the handling and turning radius required for sampling along structure and in shallow water conditions (Fig. 2). The Platform crew consisted of three people, including a boat operator, data collector, and live box operator. Each sample transect took approximately 25–30 min (5-min NS, 10-min OW transects) to sample and process fish.

Water volumes (m^3^) sampled by the Platform and Kodiak trawl were estimated using a General Oceanics mechanical flowmeter (Model 2030). The length (linear meters) of each tow was calculated by multiplying meter rotations with the Standard Speed Rotor Constant (26,874) and dividing the result by a conversion factor (999,999; Brandes & McLain, 2001). Water volume sampled was calculated by multiplying the number of linear meters traveled per tow by net mouth opening. When shallow water prevented the Platform’s net from being completely submerged, the proportion of net submerged was considered when estimating water volume sampled. Sampling included Platform and beach seine surveys in NS (depth ≤ 1.2 m; Bowen et al., 2003; Jacobson & Galat, 2006), and Platform and Kodiak trawl in OW habitats (depth > 1.5 m; Seitz et al., 2006).

### Capture efficiency

Marked fish and nearly neutrally buoyant objects have been used to study dispersal, entrainment, and capture efficiency of water and fish (e.g., Hedrick et al., 2008; Widmer et al., 2012; Kondolf & Piégay, 2016). We performed two capture efficiency studies: one comparing efficiencies across sampling methods and habitats using marked fish, and another to test the Platform sampling speed effect on capture efficiency using marked fish and nearly neutrally buoyant objects. The fish species marked depended on resident fish availability to each sampling period, relative resiliency to handling stress, and on sizes and numbers sufficient to perform investigations.

To compare the efficiency of different methods, we captured and marked 1000 juvenile bluegill sunfish (*Lepomis macrochirus*) by soaking the fish in 25 mg L^−1^ of Bismarck Brown Y (Lawler & Fitz-Earle, 1968) in two 100-L ice chests for 45 min. Marked fish were held in floating cages in the reservoir until efficiency tests were conducted. We conducted five efficiency trials for each method using 50 marked fish per trial (250 total) on 13 and 14 June 2017. For each trial, marked fish (FL mean 48.2 mm, SD 3.46) were released from 19-L buckets filled with lake water, off a 5-m jon boat, 8 m directly in front of each gear type. Safety standards prevented us from operating the release boat between the tow lines. However, Kodiak trawl tow boats are considered part of the ‘gear’ because this method employs two boats that, along with floats, lines and spreaders, herd fish into the net (Noel, 1980). Therefore, fish were released 8 m in front of the tow boats but directly in the net’s path. Each gear type sampled straight toward the released fish and sampled past the release location far enough to ensure complete coverage of habitat where marked fish had been released. New habitat was sampled during each trial to avoid fish recapture from previous releases. For the beach seine efficiency trials, the seine was deployed 15.24 m from shore. A crewmember released marked fish 8 m directly in front of the seine and then slowly walked to shore as the seine was pulled in.

Unlike beach seine and Kodiak trawl, the Platform can sample at a relatively wide range of speeds (~ 0.25–2.2 m s^−1^). To test the effect of Platform sample speed on capture efficiency we conducted 35 efficiency trials in OW (depths 5–8 m) of nearly neutrally buoyant particles (350 radishes (*Raphanus sativus*); stem and root removed; mean diameter 32.9 mm SD 3.54) and marked black crappie (*Pomoxis nigromaculatus*) (875 individuals; mean FL 41.09 mm, SD 7.82), a relatively common and available species in both habitats. Our goal was to run a minimum of 5 Platform transects (max 8) per ~ 0.5-kn (0.3 m s^−1^) increment starting at 0.5 kn and up to ~ 3 kn (~ 1.5 m s^−1^). Fish marking and release methods for the Platform speed trials were similar to the comparative efficiency study previously described.

### Fish assemblages

Fish sampling was conducted using a paired approach where samples in NS and OW habitats were collected on the same days without re-sampling the same sample sites (Fig. 1). Sample locations were chosen to represent each of the two habitat types while avoiding areas disturbed by patrons of this public reservoir. Nearshore sites were delineated and recorded using a sub-meter handheld GPS unit (Trimble® GeoXT, Trimble, Inc., Sunnyvale, CA) and identified with visible onshore markers allowing seining to occur on the left half of a sample site while the Platform simultaneously sampled the right half of the same site for the first paired sample. Sequential sampling alternated sampling orientation by gear types (i.e., seining to the right, Platform to the left of site marker). Open deep-water habitat was sampled in a similar manner, switching sides for each subsequent sampling transect with no site repetition.

Nearshore habitat was sampled by Platform and beach seine along the reservoir shoreline (12 paired samples). Seine crews further avoided areas of dense macrophyte cover, and boulders or snags that could reduce capture efficiency. The Platform operator skirted the edge of hard objects, such as rock outcroppings and tree trunks greater than approximately 0.1 m. Each Platform NS sample lasted approximately 5 min (25 min total). A target additive sampling duration of approximately 5 min for 1–3 seine hauls (average 2) per site was chosen to provide the most analogous beach seine sampling effort (measured in time) and to offer data at two spatial scales (e.g., time and volume sampled) for comparing results (LaPointe et al., 2006; Winston, 2011).

Open water sampling by Platform and Kodiak trawl was conducted along eight roughly parallel transects and at a sufficient distance to avoid disturbance from paired sampling gears (e.g., fish disturbance; approximately 50 m apart). Sites were selected for water depths of approximately 4.6–10.5 m, with minimum public boat traffic. The Kodiak trawl was towed by two boats running in parallel (Fig. 2) at approximately 0.6 m s^−1^. The Platform was operated at approximately 1.5 m s^−1^ in OW. Each OW sampling effort lasted approximately 10 min (Platform: 30 min total; Kodiak trawl: 35 min total).

All captured fish were identified to species, except black bass (*Micropterus* spp.) because black bass introductions beyond their native ranges have led to hybridization within the genus, making species determinations difficult (Whitmore & Hellier, 1988; Pierce & Van Den Avyle, 1997). Therefore, all juvenile *Micropterus* spp. were grouped as black bass. All captured fish were enumerated. Fork lengths (FL, mm) of the first 20 randomly selected individuals of each taxon and length category were measured to reduce time expended on fish processing. All captured fish were released following data collection at each capture site. Physical habitat was visually classified to estimate cover type and amount as well as dominant substrate type (Kaufmann et al., 2014).

## Data analysis

### Capture efficiency

We tested for differences in capture efficiency (the proportion of recaptured marked bluegill sunfish) among methods (i.e., beach seine, Platform NS, Platform OW and Kodiak trawl) using a Tukey–Kramer HSD test with the JMP statistical software package (JMP 5.1.2; SAS Institute Inc., Cary USA; Sall et al., 2017). Model residuals were visually inspected to ensure the assumption of homogeneity of variance was satisfied. (see Table S1).

We tested Platform sample speed effect on capture efficiency (proportion of recaptured nearly neutrally buoyant particles and marked black crappie) using a quasi-binomial generalized linear model (GLM) to account for overdispersion in recapture rate and tested for differences in recapture efficiency of particles and fish with an F-test using the JMP statistical software package (JMP 5.1.2; SAS Institute Inc., Cary USA; Sall et al., 2017).

### Fish assemblage

#### Catch-per-unit-effort

The total number of fish captured, relative taxa abundance, and water volume sampled was enumerated for each sample. To standardize catch data among gear types, we calculated catch-per-unit-effort (CPUE) by dividing catch by estimated water volume for each sample. We compared CPUE and taxa richness among sampling methods with Tukey–Kramer HSD tests (JMP 5.1.2; SAS Institute Inc., Cary, USA). Model residuals were visually inspected to ensure the assumption of homogeneity of variance was satisfied and a normal probability plot employed to test for the assumption of normality.

#### Relative abundance

We visually compared species relative abundance for each habitat and gear type by ordinating the sample-by-species-relative abundance matrix with non-metric multidimensional scaling (NMDS). The NMDS was conducted on the Bray–Curtis dissimilarity matrix from the Wisconsin double standardization and square root transformed catch data (Rabinowitz, 1975).

#### Species richness

We compared taxa richness among sampling gears within NS and OW habitats using rarefaction curves based on the number of individuals captured (Gotelli & Colwell, 2001). Individual rarefaction curves found the expected mean taxa richness from random permutations of the data. The NMDS calculates stress, which measures the goodness of fit of the Bray–Curtis similarity matrix onto the 2-dimensional NMDS plot. Fish taxa that represented < 1% of total catch for each sampling gear were excluded from the NMDS to reduce the influence of rare species on the final ordination (Legendre & Gallagher, 2001). The NMDS and species rarefaction curve analysis were performed in the program R (R development Core team 2020), using the package “vegan” (Oksanen et al., 2014). To examine potential year effects on species relative abundance, we performed an NMDS ordination using year as the only factor, without gear or habitat.

### Length frequency

We evaluated whether the FL distribution of black crappie, the most common species captured among sampling gears and habitat types, differed among gear types within NS and OW habitats during the 14 June 2017 sampling, the day when they were most frequent. To reduce potential bias introduced from sub-sampling, each subsample was extrapolated to the sample and the statistics calculated on the entire sample for each method and habitat (Bettoli & Miranda, 2001). We compared size frequency distributions among gear types within NS and OW habitats using a Kolmogorov–Smirnov test. A Bonferroni correction was applied to account for multiple comparisons (*P* < 0.05 to signify significant differences).

## Results

Nearshore habitats displayed a variety of cover types (Table 1). Sampled habitat structure was relatively similar between beach seine and Platform NS, with the exception of gravel substrate, which was seven times more prevalent at beach seine sample sites, and woody vegetation which was over 7 times more prevalent in Platform NS samples. Total cover estimates ranged from sparse to moderate in beach seine and moderate to heavy in Platform NS sites. Substrate could not be identified in OW habitat due to depth obscuring visual classification; no cover was observed in OW habitat.

**Table 1:** Summary detailing mean (SD) cover proportion of different habitat structures (cover and substrate) for the beach seine, Platform nearshore (NS). See Kaufmann et al. (2014) for classification scheme. (-) indicates insufficient sample size to calculate SD. None of the cover categories were detected in open water. Depth prohibited substrate classification in open water

Habitat structure	Type	Beach seine	Platform (NS)
Cover			
	Brush	0.02 (-)	0.0
	Grasses and forbes	0.05 (-)	0.1 (-)
	Grazed grasses and forbes	0.45 (0.2)	0.43 (0.3)
	Rock outcropping	0.0	0.05 (-)
	Woody vegetation	0.02 (-)	0.14 (0.1)
	None	0.46 (0.3)	0.28 (0.1)
Substrate			
	Silt/clay/muck	0.63 (0.3)	0.56 (0.2)
	Gravel	0.37 (< 0.1)	0.05 (0.0)
	Bedrock	0.0	0.1 (-)

### Capture efficiency

Mean capture efficiency (recapture proportion of bluegill sunfish) for all gear types was 0.20 Table S1.Capture efficiency for trials ranged from 0.02 (1 recapture) to 0.76 (38 recaptures). Mean Platform OW capture efficiency (0.40; SE 0.12) was significantly greater than mean Kodiak trawl capture efficiency (0.08; SE 0.05, Table 2). Mean beach seine capture efficiency (0.19; SE 0.05) was not significantly different than that of the Platform NS (0.17; SE 0.05). Mean Platform NS capture efficiency was greater than OW but not significantly.

**Table 2:** Comparisons of mean marked bluegill sunfish recaptures observed per sample method for all pairs (Tukey–Kramer HSD for multiple comparisons at α = 0.05). Nearshore (NS); open water (OW). Bold numbers indicate significant difference

Level	- Level	Mean Difference	Std err dif	Lower CL	Upper CL	*P* value
Platform OW	Kodiak Trawl	0.32	0.10	0.04	0.60	**0.02**
Beach seine	Platform NS	0.01	0.10	-0.27	0.29	1.00
Platform OW	Platform NS	0.23	0.1	-0.05	0.51	0.13

**Fig. 4:**
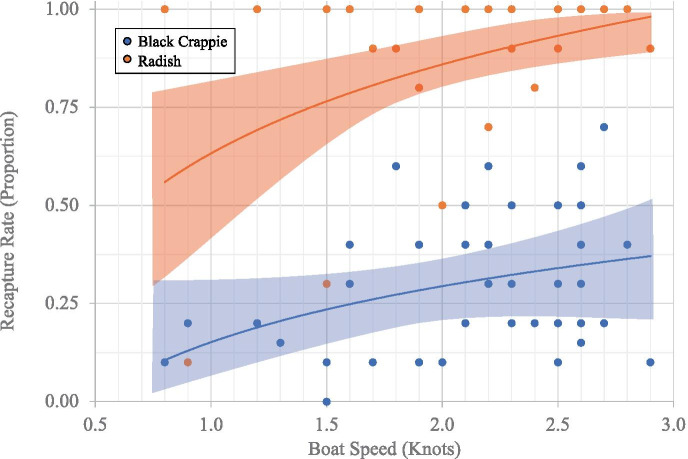
Comparison of recapture rates for semi-buoyant particles (Radish) and black crappie (Crappie) by Platform sampling method at different speeds. Fit line (polynomial, quadratic)

At speeds of 0.8–3.0 KTS (Fig. 4), the Platform was more efficient at semi-buoyant particle recapture (mean 0.86; SE 0.03) compared to fish (mean 0.30; SE 0.03) (*DF* = 76,1; *F* = 125.67; *P* < 0.01). Platform sample speed had a significant positive effect on recapture of both particles (*F* = 4.77; *DF* = 41,1; *P* = 0.035) and fish (*F* = 5.40; *DF* = 34,1; *P* = 0.027).

### Fish assemblage

Catch-per-unit-effort. Catch-per-unit-effort appeared most similar between gears within the same habitat types (Table 3). Gears in NS displayed a relatively wide spread of CPUE estimates. Open water sampling displayed a narrower band of CPUE.

**Table 3:** Summary of sample number, estimated average sample and process time, crew number, mean catch (SD), volume sampled (SE), catch-per-unit-effort (CPUE), and mean taxa per sample (SE) by beach seine, Platform nearshore and open water, and Kodiak trawl

Method	Habitat	Samples	Sample time (min)	Process time (min)	Crew	Catch	Volume (m^3^)	CPUE (catch m^−3^)	Taxa per sample (SE)
Beach seine	Nearshore	25	2.5	20	4	112.20 (22.15)	64.64 (7.21)	2.00 (0.36)	3.36 (0.29)
Platform	Nearshore	12	5.0	20	3	1504.17 (1180.76)	627.68 (39.23)	2.55 (0.90)	5.20 (0.63)
Platform	Open water	8	10.0	20	3	26.87 (39.80)	1315.51 (126.20)	0.02 (0.01)	2.00 (0.33)
Kodiak trawl	Open water	8	10.0	25	5	21.90 (9.15)	4740.80 (360.90)	0.01 (0.01)	1.40 (0.22)

When testing for differences, Platform CPUE was similar to or greater than beach seine in NS and Kodiak trawl in OW (Table 4). However, CPUE was significantly greater for NS than OW regardless of which gear was compared.

**Table 4:** Comparisons of mean catch-per-unit-effort (CPUE) observed per sample method transect in nearshore (NS) and open water (OW) for all pairs using Tukey–Kramer HSD. Bold numbers indicate significant differences between methods

Level	-Level	Difference	Std err dif	Lower CL	Upper CL	*P* value
Platform NS	Kodiak OW	2.54	0.89	0.0.22	5.98	**< 0.01**
Platform NS	Platform OW	2.54	0.90	0.21	5.95	**< 0.01**
Beach seine NS	Kodiak OW	1.99	0.35	0.18	4.23	**0.04**
Beach seine NS	Platform OW	1.98	0.35	0.18	4.23	**0.04**
Platform OW	Kodiak OW	0.01	< 0.01	0.20	3.42	0.06
Platform NS	Beach seine NS	0.55	0.54	−0.04	1.76	0.50

#### Relative abundance

A total of 13,487 fish, representing 13 taxa were sampled from NS and OW habitats between both sampling years (Table 5). In NS, the Platform collected 10,832 individuals (80% total), representing 11 taxa (85%) and the beach seine collected 2138 (16%), representing 7 taxa (54%). In OW, the Platform sampled 371 (3%) individuals representing 6 taxa (46%) and the Kodiak trawl sampled 147 individuals (1%) representing 3 taxa (23%).

**Table 5:** Summary of total catch with percent relative abundance (in parentheses) by taxon for Kodiak trawl (KT), Platform (P) and beach seine (BS) in nearshore (NS), and open water (OW) habitats

Taxa	Beach seine NS	Platform NS	Platform OW	Kodiak trawl OW		Total
Black bass *Micropterus* spp.	1427 (66.74)	488 (4.51)	3 (0.81)	2 (1.36)	1920 (14.24)	
Black crappie *Pomoxis nigromaculatus*	566 (26.47)	8823 (81.46)	82 (22.10)	76 (51.70)	9547 (70.79)	
Bluegill sunfish *Lepomis macrochirus*	31 (1.45)	709 (6.55)	1 (0.27)	0	741 (5.49)	
Common carp *Cyprinus carpio*	28 (1.31)	283 (2.61)	0	0	311 (2.31)	
Green sunfish *Lepomis cyanellus*	0	0	1 (0.27)	0	1 (< 0.01)	
Golden shiner *Notemigonus crysoleucas*	0	2 (0.02)	0	0	2 (0.01)	
Hardhead minnow *Mylopharodon conocephalus*	0	0	1 (0.27)	0	1 (< 0.00)	
Prickly sculpin *Cottus asper*	1 (0.05)	31 (0.29)	0	0	32 (0.24)	
Redear sunfish *Lepomis microlophus*	0	2 (0.01)	0	0	1 (< 0.01)	
Sacramento blackfish *Orthodon microlepidotus*	36 (1.68)	7 (0.06)	0	0	43 (0.32)	
Sacramento pikeminnow *Ptychocheilus grandis*	0	2 (0.02)	0	0	2 (0.01)	
Threadfin shad *Dorosoma petenense*	0	484 (4.47)	283 (76.28)	69 (46.94)	836 (6.20)	
Western mosquitofish *Gambusia affinis*	49 (2.29)	1 (0.01)	0	0	50 (0.37)	
Total	2138	10,832	371	147	13,487	

The most common taxa in overall catch (> 5% of total catch) were black crappie, black bass, threadfin shad (*Dorosoma petenense*), and bluegill sunfish. Black crappie and black bass were caught by all gear types; however, black bass were rare in OW sampling. Eleven taxa were captured by beach seine and Platform NS, with seven taxa represented in the catch from both gears (e.g., four taxa not shared by NS gear catches; Table 5). Platform and Kodiak trawl captured the same three taxa in relatively high numbers with three additional rare OW taxa (single observations) captured by Platform (i.e., hardhead minnow, green sunfish, bluegill sunfish).

#### Species richness

**Fig. 5:**
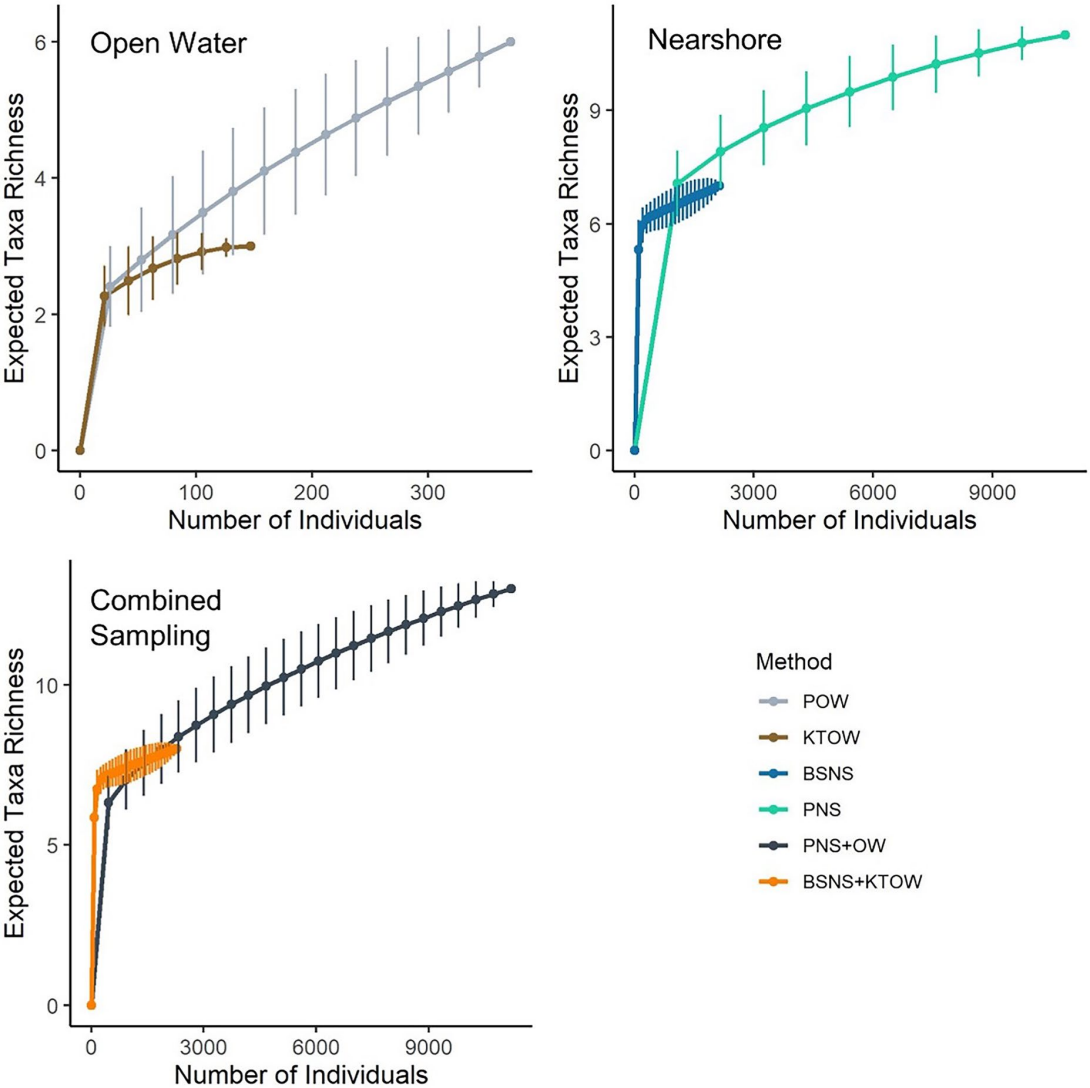
Individual rarefaction curves and standard deviation for Platform (POW) and Kodiak trawl (KTOW) in open water and beach seine (BSNS) and Platform (PNS) in nearshore and combined sampling methods in both habitats (PNS + OW and BSNS + KTOW). Note different axes

Mean number of taxa per sample was higher in NS than OW and higher in Platform samples than the other two methods used in both habitats (Table 6). Based on individual rarefaction curves, the Platform was able to detect more taxa as the number of sampled individuals increased in each habitat and when sampling methods were combined (Fig. 5).

**Table 6:** Comparisons of mean taxa observed for nearshore (NS) and open water (OW) per sample method transect for all pairs using Tukey–Kramer HSD. Bold numbers indicate significant differences between methods

Level	- Level	Difference	Std err dif	Lower CL	Upper CL	*P* value
Platform NS	Kodiak OW	3.80	0.57	2.267	5.33	**< 0.01**
Platform NS	Platform OW	3.20	0.57	1.67	4.73	**< 0.01**
Beach seine NS	Kodiak OW	1.96	0.53	0.54	3.37	**< 0.01**
Platform NS	Beach seine NS	1.84	0.53	0.43	3.26	**< 0.01**
Beach seine NS	Platform OW	1.36	0.53	− 0.06	2.77	0.07
Platform OW	Kodiak OW	0.60	0.57	− 0.93	2.13	0.72

For OW, the Platform rarefaction curve climbed above Kodiak trawl when samples surpassed ~ 20 individuals. Kodiak trawl rarefaction curves appeared to asymptote at ~ 100 individuals, whereas the Platform rarefaction curve continued to gain taxa richness as the number of individuals sampled in OW increased. As NS sampled fish increased to ~ 150, the expected taxa richness for Platform was approximately four, while beach seine was three. The Platform rarefaction curve climbed above the beach seine when samples surpassed 1000 individuals. As the number of individuals increased to ~ 2000, the expected Platform taxa richness was approximately eight, while beach seine was seven. Neither rarefaction curve for NS sampling appeared to asymptote as more individuals were sampled. For sampling across habitats, the Platform (PNS + POW) was slightly more efficient when sampling > 2000 individuals than the beach seine and Kodiak trawl combined (BSNS + KTOW). However, the Platform’s rarefaction curve appeared to climb at a higher rate when compared to the beach seine and Kodiak trawl and under similar sampling effort (total time).

**Fig. 6:**
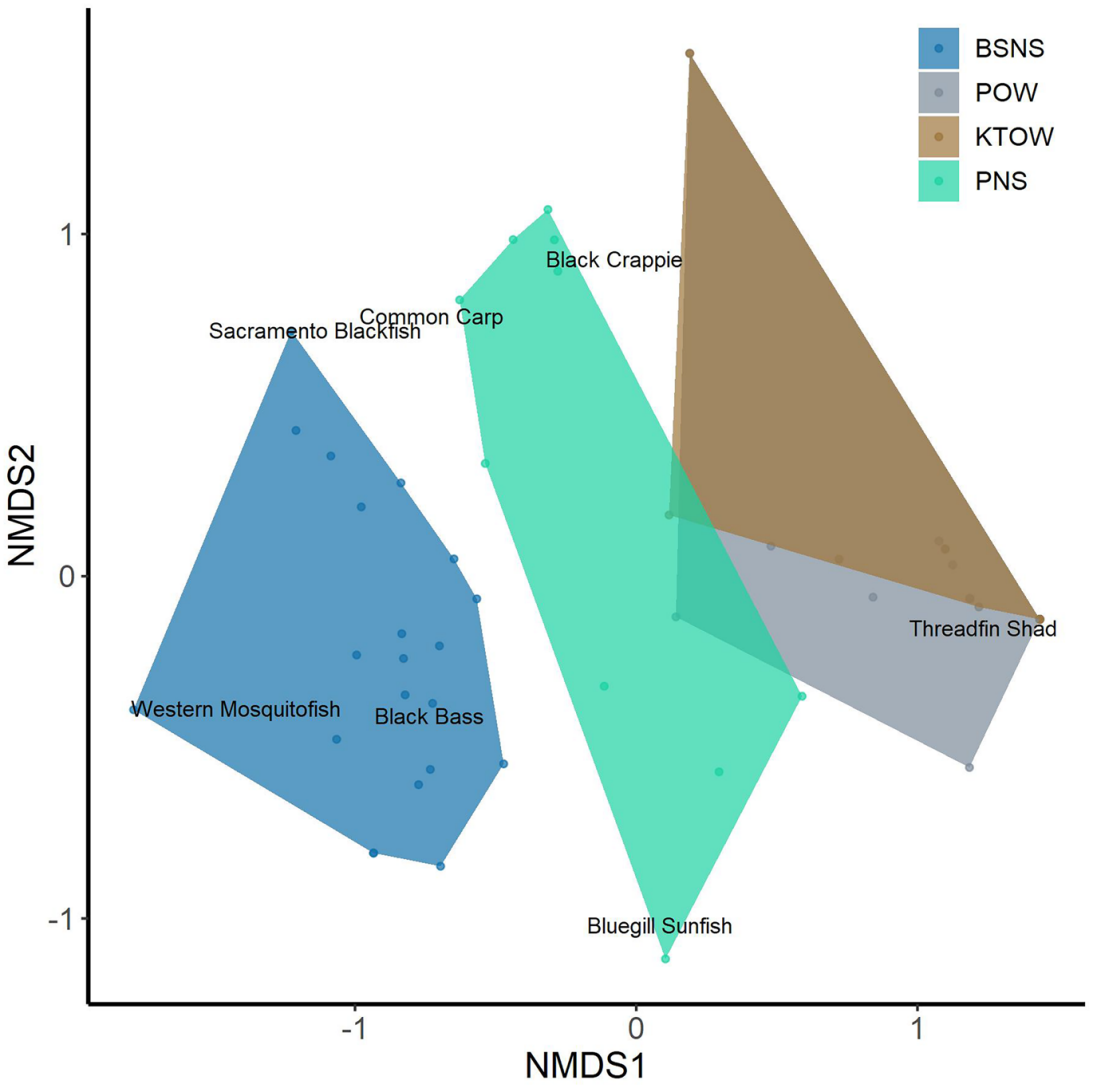
Nonmetric multidimensional scaling (NMDS) plot of fish community data collected via Kodiak trawl (KT), Platform (P), open water (OW), and Platform and beach seine (B) nearshore (NS)

Resulting biplots from the NMDS displayed substantial overlap between sampling years; therefore, we combined years to increase sampling power (Fig. 6). The combined year NMDS ordination had a stress value of 0.10, indicating rank-order distances in the original community matrix were preserved in the NMDS ordination and the fit was sufficient.

The beach seine sample score polygons were clustered apart from other methods and were negatively correlated with axis 1. Fish taxa associated with beach seine polygon position were Western mosquitofish (*Gambusia affinis*), black bass, and Sacramento blackfish (*Orthodon microlepidotus*). The Platform NS sample score polygon was closest in proximity to beach seine NS polygon along the axis 1, clustered around zero but were more similar to OW communities based on polygon overlap. Fish taxa associated with Platform NS position were black crappie and bluegill, with common carp (*Cyprinus carpio*) intermediate between beach seine and Platform polygons for NS. Open water fish communities sampled by Kodiak trawl and Platform were positively correlated with axis 1 and similar based on their overlap. Fish taxa associated with OW Kodiak trawl and Platform polygon positions were mainly threadfin shad, with black crappie intermediate between OW sampling gear and Platform NS.

Greater relative abundance of threadfin shad and lower relative abundance of black bass in Platform NS catch likely accounted for the majority of dissimilarity with beach seine catch. Higher Platform catch of threadfin shad accounted for most of the dissimilarity with Kodiak trawl in OW.

The higher number of taxa sampled by the Platform NS and differences in black crappie and threadfin shad relative abundances likely accounted for the majority of the dissimilarity between NS and OW sampled by the Platform.

### Fish size frequency

**Fig. 7:**
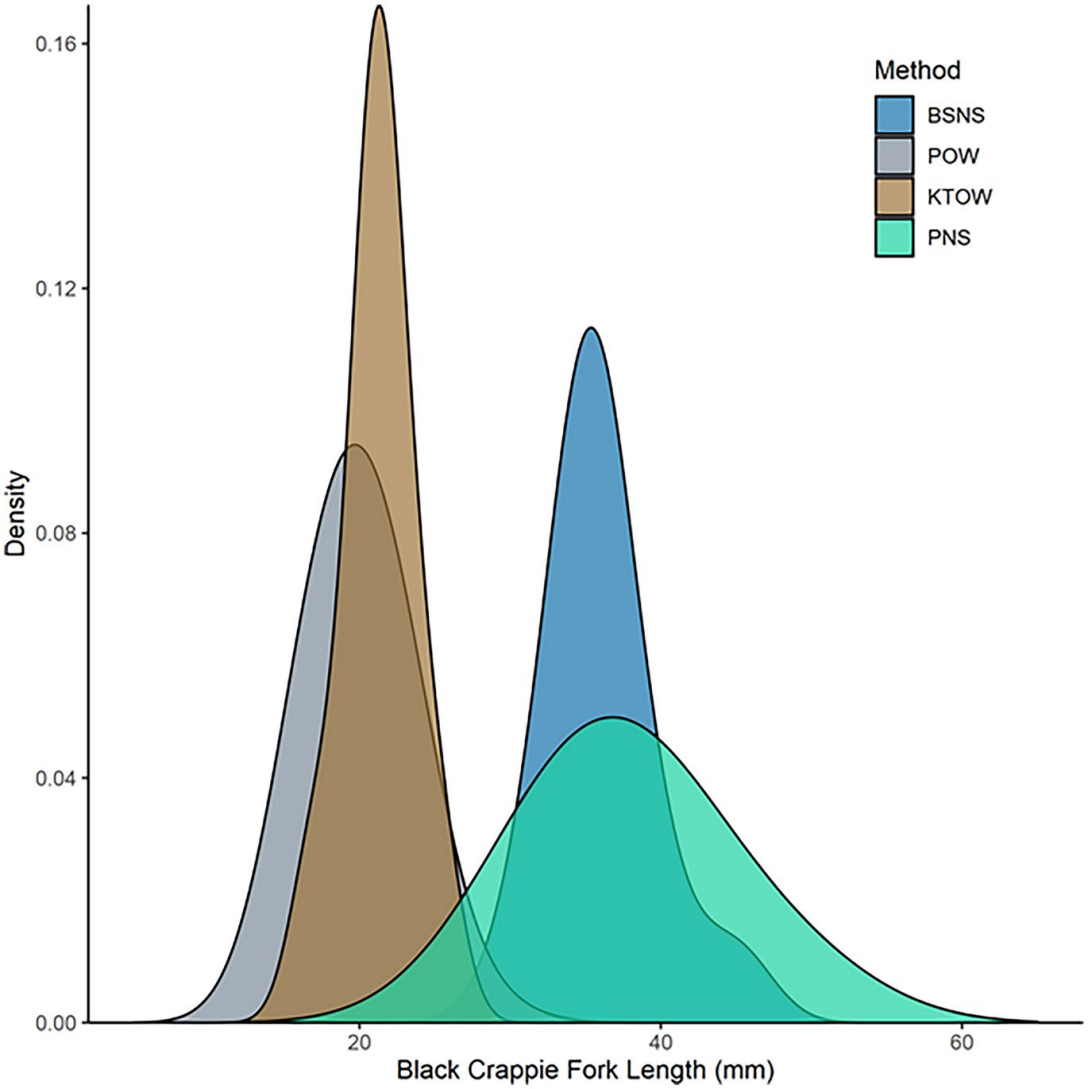
Fork length density distribution of black crappie captured by the Kodiak trawl and Platform in open water (OW) and beach seine and Platform in nearshore (NS) habitat

Black crappie was the most common shared (relative abundance > 0.22) taxon by gear and habitat type (catches; Table 5). In NS habitat, distribution of black crappie FL captured by beach seine (36.1 mm ± 3.39 SD) and Platform (37.8 mm ± 5.81 SD) was not significantly different (Kolmogorov–Smirnov test: *D* = 0.25, Bonferroni corrected *P* = 0.57; Fig. 7). However, black crappie FL distribution captured by Kodiak trawl (21.3 mm ± 2.23 SD) was significantly greater than the Platform (19.9 mm ± 2.96 SD) in OW (Kolmogorov–Smirnov test: *D* = 0.33, Bonferroni corrected *P* = 0.011). The distribution of black crappie FL caught by beach seine was significantly different than that captured by Kodiak trawl (Kolmogorov–Smirnov test: *D* = 1, Bonferroni corrected *P* < 0.001) and the distribution of black crappie FL caught by Platform in NS was significantly different than OW (Kolmogorov–Smirnov test: *D* = 1, Bonferroni corrected *P* < 0.001).

## Discussion

Gear-specific characteristics can bias fish catch data to varying degrees, based on relationships between gear attributes and how effectively and representatively a gear samples a given habitat type or species. These biases complicate attempts to describe habitat-specific abundance and ontogenetic or behavioral shifts of species between distinct habitat types within lentic ecosystems. Pierce et al. (1990) reported snagging and seine rolling can reduce capture efficiencies and that benthic fishes are less susceptible to seining than midwater fishes. Likewise, physical complexity including rocks, macrophytes, and tree branches can provide fish escape routes during seining (Lyons, 1986). In our study, the seine crew sampled in areas of relatively smooth bank transition with sparce vegetation, generally avoiding features thought to roll or snag nets (e.g., rock outcroppings; woody vegetation). Alternatively, the Platform effectively sampled through woody vegetation when plants were flexible (e.g., trunks and branches less than ~ 0.1 m), articulated in the summarization of sampled cover types. During Platform NS operation, we broke two shear pins: one impacting a bedrock out cropping and the other a concrete mooring. Even so, we were able to quickly replace the pins (e.g., < 2 min) and continue sampling. Due to the solid net frame and frame base wheels, the Platform net mouth remained relatively close to the lakebed in NS habitat (e.g., < 10 cm; no net rolling, snagging or lifting during testing). This may explain the greater presence of prickly sculpin, a benthic species, in Platform samples (Anderson, 1985). Clear differences in substrate cover sampled by the two NS methods articulate avoidance of specific features by the seine crew informed by previous research not available for the Platform (Bonar et al., 2009). Therefore, these results should be considered cautiously and warrant future research.

The Platform also sampled a greater water volume than the beach seine during sampling events of similar duration, due to increased sampling speed. The beach seine could sample completely to shore (i.e., zero depth) as long as the shoreline was relatively smooth, whereas the Platform effectively sampled waters deeper than 0.4 m. In OW, the Kodiak trawl sampled a greater water volume than the Platform under a comparable time period, but was less maneuverable and therefore could not sample complex habitats or shallower than approximately 2 m. These results suggest the Platform can effectively sample in both NS and OW habitats and generate comparable abundance estimates from both habitat types. Future research focused on comparison with other offshore techniques for sampling deeper in the water column (> 3 m; e.g. benthic trawls, gill nets), cost-effectiveness, including staffing time, operation cost, and data analysis may further inform Platform value to fisheries research (Collins et al., 2017).

In general, the Platform had higher recapture efficiency than the other two methods. This is likely related to sampling speed because boat speed had a significant effect on Platform recapture success for both particles and marked fish. However, Platform recapture efficiency of bluegill sunfish was lower than for particles. These data suggest that even for inanimate objects, diffusion generally influences sampling particles, whether inanimate or free-swimming, in a set water volume. Thus, the slower the sample method, the longer time particles have to diffuse. This, coupled with fish avoidance behavior, demonstrates sampling speed effect on recapture rates. A positive aspect of the Platform is that speed can be adjusted for different habitats. During this study, we sampled at speeds up to ~ 4.25 kn (2.2 m s^−1^). Visual comparison of Platform recapture efficiency reported here suggests that speeds in excess of ~ 2 kn allows for more efficient sampling of common spring-time small-bodied fish (e.g., juvenile black crappie). Fish react strongly to visual components of demersal trawls and typically perform a “fountain maneuver” whereby they are herded either towards the trawl entrance or away by visual perception of trawl boats, ropes, floats, doors, and netting at the trawl front (Wardle, 1993). It is important to acknowledge that our Kodiak trawl release strategy was complicated by safety protocol and that the greater distance from the trawl may have allowed greater avoidance response for marked fish, even with herding by the lead boats (Noel, 1980). Nevertheless, lower Kodiak trawl CPUE than the other two methods supports our observations. Future research is warranted in this area.

Another observation was differences in bluegill sunfish recapture efficiency by habitat sampled by Platform. While not statistically significant, we recaptured fewer bluegill sunfish NS than in OW. This may be due to fish behavior where bluegill sunfish may choose to evade capture by NS cover use, whereas structural cover is not generally available in OW (Savino & Stein, 1989; this study). Additionally, water displacement by Platform in NS environments may be more detectible by the Bluegill lateral line, allowing capture evasion (Windsor & McHenry, 2009).

Catch-per-unit-effort was most similar between gear types within the same habitat. Sampling in NS displayed a wide CPUE range, with beach seine means similar to those of the Platform. Open water CPUE demonstrated less variability than NS CPUE and centered around zero with Platform averages similar to Kodiak trawl. Variation in CPUE among individual samples suggests fish were patchily distributed within the reservoir, potentially having a large influence on CPUE variation for specific habitat-gear type combinations. Nonetheless, our study indicates the Platform can detect fish CPUE differences related to habitat type (NS and OW) whereas typically, two gears would need to be employed, complicating quantitative comparisons among habitat types.

The high between-sample variation suggests more samples were needed for more robust statistical comparisons. This study was intended to provide a preliminary comparison of the Platform to traditional sampling gears. Future evaluations should include sufficient sample sizes to strengthen statistical comparisons and a wider range of common fish sampling techniques (e.g., Jurajda et al., 2009). Regardless, these data provide compelling evidence of the Platform’s ability to adequately sample small-bodied fish across habitat types that would otherwise require multiple gear types.

This study showed differences in fish assemblage structure between OW and NS habitats. Together, these data suggest that unlike the conventional individual sampling methods we tested, the Platform can detect important fish assemblage differences associated with distinct habitat types and can provide standardized data to support comparative assessments across habitats. In OW, the relative abundance of taxa and NMDS polygon location were similar between Platform and Kodiak trawl. In contrast, NS communities collected by Platform and beach seine were more divergent. This was primarily due to relatively more black crappie and threadfin shad captured by the Platform NS. Although threadfin shad are more typically associated with the pelagic environment, both species can feed in OW that the beach seine cannot effectively sample, or can flee when crews deploy seines at the deep end of NS habitats (Burns, 1966; Ehlinger & Wilson, 1988). Conversely, many of the black bass observed in NS habitat were captured in relatively shallow water (less than ~ 0.15 m), potentially making them more susceptible to the beach seine than Platform. The relatively small sample sizes collected during this study may have increased the possibility of dissimilarities between beach seine and Platform due to random collections of rare taxa or unique behaviors of fish taxa encountered with each method or microhabitat use by species. The differences in relative abundance observed between NS and OW habitats sampled by the Platform were consistent with general trends of greater abundance and taxa diversity in NS than OW habitats (Angermeier & Karr, 1984; Scott & Angermeier, 1998; Hudon et al., 2000; Brooks et al., 2004; Willis et al., 2005). All sampling methods are selective based on the interaction between gear characteristics and species’ behavior and descriptors of the fish assemblage are always estimated with error. Overall, the Platform NS was able to collect the same fish taxa encountered in the beach seine.

The Platform collected a greater number of fish in a similar amount of sampling time relative to other methods, which influenced taxa richness estimates. In OW habitat, the Platform collected more than double the number of individuals and species as the Kodiak trawl. Rarefaction indicated richness estimates with the Kodiak trawl began to asymptote at ~ 150 individuals and 3 taxa whereas richness estimated by the platform was still climbing at ~ 350 individuals collected from 6 taxa. Similarly, in NS habitat the Platform captured > 3 times the number of individuals and 4 more taxa than the beach seine with half the samples performed. This pattern suggests that because the Platform can collect more individuals in the same amount of time relative to the trawl or seine, it may be more effective at detecting species that are in low abundance such as threatened or endangered species and non-native species that have recently invaded (Mitchell et al., 2017).

Greater fish numbers and taxa were observed in NS than OW habitats, either by separate methods or by Platform alone. Nearshore reservoir habitats often provide greater cover, primary productivity, and food availability than OW habitats due to light penetration to the substrate, contributing to greater fish abundance and diversity (Hudon et al., 2000; Taniguchi et al., 2003; Thomaz et al., 2008). Therefore, complexity differences observed between NS and OW in this study may have contributed to greater fish abundance and diversity in NS habitats (Gratwicke & Speight, 2005; St. Pierre & Kovalenko, 2014; Consoli et al., 2016).

Unlike the beach seine and Kodiak trawl, the Platform is specifically designed to collect comparable environmental and biological data across habitat types and representatively sample NS and OW habitats, which is useful for detecting ontogenetic or behavioral habitat shifts. Our comparison of black crappie FL captured by each gear type demonstrated distinct fish size differences between Camanche Reservoir habitats. In particular, juveniles were smaller in OW than NS samples. Post et al. (1995) found the duration of age-0 black crappie pelagic residence can range from two to six weeks before transitioning to the littoral zone in a eutrophic Wisconsin lake. Similarly, Weber & Brown (2012) found that bluegill sunfish and black crappie in OW offshore habitat were smaller compared to those captured in nearshore habitats, suggesting a size-related nearshore migration may have occurred. Our results were consistent with these findings, suggesting the Platform accurately detected ontogenetic shifts in habitat use by a common lentic species observed in this study. It is important to note that fish size differences we observed by beach seine and Kodiak trawl may have not represented true population differences due to gear selectivity (Binion et al., 2009; Fischer & Quist, 2014a, 2014b).

This study implies the Platform can successfully collect data on small-bodied fish and juveniles of larger fish that are equally or more informative than those collected by using multiple gear types such as beach seine and Kodiak trawl that are difficult to compare directly. Our study revealed quantifiable differences in fish CPUE, distribution (nearshore vs. offshore), taxa richness, and size in NS and OW habitats sampled with different gears in a eutrophic reservoir. Furthermore, based on marked fish recapture rates we suggest that Platform gear bias was relatively consistent between NS and OW habitats, and much more consistent than bias associated with different gears. Thus, the Platform appears to directly address the need to collect comparable individual fish and fish community data from different habitats while reducing gear bias associated with using different gears across habitat types. While we most likely did not sample all species available within the reservoir at the time of our study, the Platform enabled standardized habitat use comparisons by the small-bodied fish community and individual target species. Such empirical findings can help refine our understanding of general ontological shifts in fish habitat use, and assessment of habitat management actions such as restoration, rehabilitation, and alternative flow regimes in regulated systems (Gibson et al., 2003; McElroy et al., 2018).

## Conclusions

The Platform appears to provide a single, integrated, efficient method for sampling small-bodied fish assemblages across NS and OW habitats while characterizing a series of associated biotic and abiotic habitat attributes. It offers an exciting new fisheries science tool for monitoring lentic fish communities while providing a more informative ecological context for data interpretation than two traditional sampling methods. Although the current Platform design effectively sampled small-bodied fish across shallow NS and deep OW habitats at depths from 0.4 to 3 m, modification of the net attachment angle along with a greater net height could potentially extend the depth range of sampling without significantly reducing capture efficiencies. The maximum depth the Platform could be designed to sample should be studied further. Future Platform surveys, including comparisons against different methods, in additional water bodies with different habitat attributes and fish communities are expected to better characterize the benefits, limitations, and range of applications for this innovative, multi-purpose fisheries and habitat evaluation tool.

## Supplementary Information

Below is the link to the electronic supplementary material.

Supplementary file1 (DOCX 14 KB)

## Data Availability

We will upload to journal website.
