# Supplementary material for: Comparison of three sampling methods for small-bodied fish in lentic nearshore and open water habitats
Source: Environ Monit Assess. 2021 Apr 9;193(5):255. doi: 10.1007/s10661-021-09027-9 (PMC8035114; doi:10.1007/s10661-021-09027-9)
Supplement: Supplementary file 1 — Supplementary file1 (DOCX 14 KB) [file 10661_2021_9027_MOESM1_ESM.docx]

**Supplement Information**

Table S1. Number of recaptured marked Bluegill Sunfish with (proportion of total release) by method from 50 released in each of five trials. Nearshore (NS); Open water (OW).

| **Method** | **Trial 1** | **Trial 2** | **Trial 3** | **Trial 4** | **Trial 5** |
| --- | --- | --- | --- | --- | --- |
| Beach Seine | 14 (0.28) | 7 (0.14) | 5 (0.10) | 3 (0.06) | 15 (0.30) |
| Platform (NS) | 17 (0.34) | 6 (0.12) | 2 (0.04) | 11 (0.22) | 6 (0.12) |
| Platform (OW) | 11 (0.22) | 38 (0.76) | 11 (0.22 | 10 (0.2) | 29 (0.58) |
| Kodiak Trawl | 6 (0.12) | 4 (0.08) | 2 (0.04) | 1 (0.02) | 6 (0.12) |
